# The Pro-Inflammatory Chemokines CXCL9, CXCL10 and CXCL11 Are Upregulated Following SARS-CoV-2 Infection in an AKT-Dependent Manner

**DOI:** 10.3390/v13061062

**Published:** 2021-06-03

**Authors:** Victoria Callahan, Seth Hawks, Matthew A. Crawford, Caitlin W. Lehman, Holly A. Morrison, Hannah M. Ivester, Ivan Akhrymuk, Niloufar Boghdeh, Rafaela Flor, Carla V. Finkielstein, Irving Coy Allen, James Weger-Lucarelli, Nisha Duggal, Molly A. Hughes, Kylene Kehn-Hall

**Affiliations:** 1 National Center for Biodefense and Infectious Diseases, School of Systems Biology, George Mason University, Manassas, VA 20110, USA; vcallah@gmu.edu (V.C.); nboghdeh@masonlive.gmu.edu (N.B.); mflor.rafaela@gmail.com (R.F.); 2 Department of Biomedical Science and Pathobiology, Virginia-Maryland College of Veterinary Medicine, Virginia Polytechnic Institute and State University, Blacksburg, VA 24060, USA; sah1026@vt.edu (S.H.); Woodsonc@vt.edu (C.W.L.); hamorrison18@vt.edu (H.A.M.); Iakhrymu@vt.edu (I.A.); icallen@vt.edu (I.C.A.); weger@vt.edu (J.W.-L.); nduggal@vt.edu (N.D.); 3 Division of Infectious Diseases and International Health, Department of Medicine, University of Virginia, Charlottesville, VA 22908, USA; mac5ee@virginia.edu (M.A.C.); mah3x@virginia.edu (M.A.H.); 4 Graduate Program in Translational Biology, Medicine and Health, Virginia Polytechnic Institute and State University, Roanoke, VA 24061, USA; hivester@vt.edu; 5 Integrated Cellular Responses Laboratory, Department of Biological Sciences and Center for Drug Discovery, Fralin Biomedical Research Institute, Virginia Polytechnic Institute and State University, Roanoke, VA 24016, USA; finkielc@vt.edu; 6 Virginia Tech Carilion School of Medicine, Virginia Polytechnic Institute and State University, Blacksburg, VA 24016, USA; 7 Center for Zoonotic and Arthropod-borne Pathogens, Virginia Polytechnic Institute and State University, Blacksburg, VA 24060, USA

**Keywords:** SARS-CoV-2, betacoronavirus, CXCL10, ARDS, ALI, cytokine storm, Calu-3, hACE2 mice

## Abstract

Severe acute respiratory syndrome coronavirus 2 (SARS-CoV-2) is a highly transmissible RNA virus that is the causative agent of the Coronavirus disease 2019 (COVID-19) pandemic. Patients with severe COVID-19 may develop acute lung injury (ALI) or acute respiratory distress syndrome (ARDS) and require mechanical ventilation. Key features of SARS-CoV-2 induced pulmonary complications include an overexpression of pro-inflammatory chemokines and cytokines that contribute to a ‘cytokine storm.’ In the current study an inflammatory state in Calu-3 human lung epithelial cells was characterized in which significantly elevated transcripts of the immunostimulatory chemokines CXCL9, CXCL10, and CXCL11 were present. Additionally, an increase in gene expression of the cytokines IL-6, TNFα, and IFN-γ was observed. The transcription of CXCL9, CXCL10, IL-6, and IFN-γ was also induced in the lungs of human transgenic angiotensin converting enzyme 2 (ACE2) mice infected with SARS-CoV-2. To elucidate cell signaling pathways responsible for chemokine upregulation in SARS-CoV-2 infected cells, small molecule inhibitors targeting key signaling kinases were used. The induction of CXCL9, CXCL10, and CXCL11 gene expression in response to SARS-CoV-2 infection was markedly reduced by treatment with the AKT inhibitor GSK690693. Samples from COVID-19 positive individuals also displayed marked increases in CXCL9, CXCL10, and CXCL11 transcripts as well as transcripts in the AKT pathway. The current study elucidates potential pathway specific targets for reducing the induction of chemokines that may be contributing to SARS-CoV-2 pathogenesis via hyperinflammation.

## 1. Introduction

Severe acute respiratory syndrome coronavirus 2 (SARS-CoV-2), is an emergent coronavirus first identified as causing widespread pneumonia in Wuhan, China in late 2019 [1,2,3]. The etiologic cause of the widespread pneumonia was identified to be SARS-CoV-2 from bronchoalveolar lavage fluid (BALF) of three patients in December 2019 [1,3]. The novel coronavirus rapidly spread via human–human transmission and by March 2020 had infected over 300,000 people in 195 countries, ultimately eliciting a declaration of a world-wide pandemic by the World Health Organization (WHO). Since the outbreak of SARS-CoV-2 the pandemic has been designated the Coronavirus disease 2019 (COVID-19) pandemic. According to the WHO, as of 26 April 2021, over 146 million people have been infected worldwide and over 3.1 million people have succumbed to the disease.

SARS-CoV-2 is a novel, emergent *Betacoronavirus* related to SARS-CoV and MERS-CoV. SARS-CoV-2 shares about 80% sequence identity with SARS-CoV and about 50% sequence identity with MERS-CoV [1,4,5,6]. SARS-CoV, MERS-CoV, and SARS-CoV-2 are enveloped, positive-sense-single-stranded RNA viruses that have a large RNA genome that ranges from 26 to 32 kilobases (kb) in length [2]. The SARS-CoV-2 genome is 29.9 kb in length and contains 16 nonstructural proteins, 4 structural proteins, and 14 open reading frames [1,2,4,7]. The SARS-CoV-2 viral envelope is composed of envelope proteins and membrane proteins that are embedded in a phospholipid bilayer that encapsulates the viral nucleocapsid. The nucleocapsid is composed of a phosphorylated nucleocapsid (N) protein that interacts directly with the viral genome [6]. Within the envelope, viral spike proteins are embedded: spike (S) proteins are arranged in trimer spikes [1,4]. The S protein is responsible for binding host-cell receptors to promote viral infection [1,4,7]. The viral S protein, S1 subunit, contains a receptor-binding domain (RBD) that is compatible with the host-cell receptor angiotensin-converting enzyme 2 (ACE-2) [1,4,7]. Additional entry factors, transmembrane protease serine 2 (TMPRSS2), and cathepsins mediate the fusion of viral and host membranes in order to promote endosomal entry mechanisms [4].

COVID-19 patients may exhibit upper respiratory symptoms, non-respiratory symptoms, or may be clinically asymptomatic [6,8]. Due to immune infiltration of the lungs as result of a ‘cytokine storm,’ patients may develop alveolar damage, acute lung injury (ALI), and acute respiratory distress syndrome (ARDS) [1,9,10,11]. MERS, SARS, and COVID-19 share features of a hyper-inflammatory state and associated disease pathologies are correlated with poor clinical outcome [12,13,14,15]. As previously recorded during other severe *Betacoronavirus* outbreaks of MERS-CoV and SARS-CoV, the development of ALI and ARDS are also common clinical features following viral infection [9,16]. Key features of the SARS-CoV, MERS-CoV, and SARS-CoV-2 infections include commonalty of hyperinflammation and high levels of the chemokine CXCL10 (also referred to as interferon inducible protein-10; IP-10), an immunostimulatory host chemokine responsible for the recruitment of a variety of immune cells to sites of infection through interaction with its cellular receptor CXCR3. In studies conducted by Huang et al., a pro-inflammatory state was observed in the serum of SARS-CoV-2 patients admitted to the intensive care unit with elevated plasma levels of CXCL10 [15]. Amongst those 41 patients in the study, 13 were admitted to the ICU with severe clinical manifestations of ARDS and elevated levels of plasma cytokines/chemokines including CXCL10 and TNFα, amongst others [15]. Additionally, in human studies in which bronchoalveolar lavage fluid (BALF) and peripheral mononuclear blood cells (PBMC) were acquired from infected patients, RNA-sequencing data revealed significantly increased transcriptional expression of *CXCL10*, together with the related CXCR3 chemokine ligands *CXCL9* and *CXCL11*, in both BALF and PBMCs as compared to healthy controls [17]. In earlier studies conducted with SARS-CoV and MERS-CoV, the enhanced expression of *CXCL10* was also observed in patient samples and correlated with characteristics of increased disease severity; pulmonary inflammation and lung damage [13,14,15].

Studies have identified the CXCL10-CXCR3 signaling axis as a major contributor to neutrophil mediated lung injury and the development of both viral and nonviral ARDS [18]. Moreover, CXCL10 is considered a key immune event contributing to the cytokine storm observed in COVID-19 patients and a potential predictive factor of clinical outcome [19,20]. Towards understanding the mechanisms by which SARS-CoV-2 triggers destructive pulmonary inflammation, the current study investigates the expression of pro-inflammatory chemokines associated with SARS-CoV-2 infection in both in vitro and in vivo models. The expression of host mediators that induce chemokine induction was also determined. Finally, the contribution of upstream signaling cascades to *CXCL9*, *CXCL10*, and *CXCL11* transcription was determined through the use of small molecule kinase inhibitors.

## 2. Materials and Methods

### 2.1. Cell Culture

Human lung epithelial cells (Calu-3) were acquired from American Type Culture Collection (ATCC, HTB-55). Calu-3 cells were maintained in 50% Ham’s-F12 medium and 50% Dulbecco’s modified Eagle medium (DMEM) supplemented with 10% fetal bovine serum (FBS), 1% L-glutamine, 1% penicillin/streptomycin, 1% sodium pyruvate and 1% nonessential amino acids. Vero (ATCC, CCL-81) cells were maintained in DMEM supplemented with 10% FBS, 1% L-glutamine, and 1% penicillin/streptomycin. All cells were maintained at 37 °C with 5% CO_2_.

### 2.2. SARS-CoV-2 Virus and In Vitro Infections

SARS-Related Coronavirus 2, Isolate USA-WA1/2020 (Cat #. NR-52281) stock virus was obtained from BEI resources and maintained at biosafety level-3 (BSL-3) and used throughout this study. All experiments employing SARS-CoV-2 were conducted using BSL-3 precautions/facilities and were approved by George Mason University’s or Virginia Tech’s Institutional Biosafety Committee.

For viral time-course experiments, Calu-3 cells (1.5 × 10^5^ per well) were plated in 24-well cell-culture plates and incubated overnight. The next day, cells were infected at a multiplicity of infection (MOI) of 1 for 1 h at 37 °C and rocked every 15 min. The cells were then washed with phosphate-buffered saline (PBS), and cell growth medium was added back to the cells. Culture supernatants and cells were collected at 1, 3, 9, 12, 18, 24, 48 and 72 h post infection (hpi) for further study. Supernatants were stored at −80 °C prior to use in endpoint analyses. Cells were collected in TRIzol™ LS reagent (Thermo Fisher Scientific) and stored at −80 °C until RNA isolation was conducted.

### 2.3. RNA Isolation and RT-qPCR

Mock-infected or SARS-CoV-2-infected Calu-3 cell lysates were subjected to RNA extraction with the Direct-zol RNA Miniprep kit (Zymo) according to the manufacturer’s instructions. Reverse transcription quantitative PCR (RT-qPCR) was performed using the StepOnePlus™ Real-Time PCR System ThermoFisher Scientific, Waltham, MA USA). TaqMan Gene Expression Assays were used for in vitro host-gene expression analysis: *CXCL9* (Hs00171065_m1), *CXCL10* (Hs00171042_m1), *CXCL11* (Hs00171138_m1), *IL-6* (Hs00174131_m1), *TNFα* (Hs00174128_m1), *IFN-γ* (Hs00989291_m1), *18S rRNA* (pan-eukaryotic) (Hs99999901_s1), and *Glucuronidase-beta* (GusB) (Hs99999908_m1). Host-gene expression analysis was conducted with the TaqMan™ RNA-to-CT™ 1-Step Kit by ThermoFisher Scientific. Fold change was calculated relative to the *GusB* or *18S RNA* endogenous control and normalized to mock samples using the ΔΔCt method.

Detection of viral RNA in Calu-3 cells was determined by RT-qPCR with use of Invitrogen’s RNA UltraSense™ One-Step Quantitative RT-PCR system. Primers were acquired from Integrated DNA Technologies and were identical to those referenced by Center for Disease Control and Prevention (CDC): SARS-CoV-2 primer pair-1, forward 5′-GAC CCC AAA ATC AGC GAA AT-3′ (2019-nCoV_N1-F) and reverse 5′-TCT GGT TAC TGC CAG TTG AAT CTG-3′ (2019-nCoV_N1-R). The primers target the *N* gene of the SARS-CoV-2 viral genome. Absolute quantification was done using StepOne software v2.3 based on the threshold cycle relative to the standard curve. The standard curve was determined using serial dilutions of SARS-CoV-2 RNA at known concentrations; standard RNA was acquired from BEI resources (Cat. #NR-52358).

### 2.4. In Vivo hACE2 Mouse Studies

All animal experiments were carried out in animal biosafety level 3 (ABSL-3) facilities at the Infectious Disease Unit (IDU) at Virginia Tech in accordance with the National Research Council’s Guide for the Care and Use of Laboratory Animals following Virginia Tech approved IACUC protocols. Female K18-human angiotensin converting enzyme 2 (hACE2) (Jackson Laboratories, B6.Cg-Tg(K18-ACE2)2Prlmn/J) were infected with 10^5^ PFU of SARS-CoV-2 via the intranasal route. A group of 5 mice was followed for survival. Additional groups of mice were serial sacrificed at 1, 2, 3, 4, and 5 dpi for organ collection (N = 5 mice each day). Uninfected mice (day 0) were used as controls (N = 5). Three mice from the survival group were euthanized on day 5 due to weight loss and were subsequently included for downstream organ analysis, resulting in the 5 dpi group being N = 8. Mouse lungs were collected in TRIzol™ LS reagent (ThermoFisher Scientific, Waltham, MA USA), homogenized, and subjected to RNA extraction techniques similar to those described above. Following extraction, RNA samples were subjected to RT-qPCR using the TaqMan™ RNA-to-CT™ 1-Step Kit by ThermoFisher Scientific. TaqMan Gene Expression Assays were used for host-gene expression analysis: *CXCL10* (Mm00445235_m1), *CXCL9* (Mm00434946_m1), *IL-1β*, *TNFα* (Mm00443258_m1), *IL-6* (Mm00446190_m1), *IFN-γ* (Mm01168134_m1), and *18S rRNA* (pan-eukaryotic) (Hs99999901_s1). Fold change was calculated relative to 18S and normalized to samples from uninfected mice using the ΔΔCt method. Viral RNA levels were determined as described above.

### 2.5. Viral Plaque Assay

Crystal violet plaque assays were performed to determine viral titers from supernatants collected from in vitro experiments and supernatants collected from homogenized lung tissue from in vivo experiments. Infected supernatants were serially diluted in phosphate buffered saline (PBS) with 1% FBS and added to confluent 6-well plates of Vero cells. Cells were incubated with serially diluted viral inoculum at 37 °C for 1 h, with rocking every 15 min. After infection, 2 mL of a 1:1:1 mixture of supplemented Minimum Essential Eagle Medium (EMEM), supplemented DMEM and 1.5% agarose in deionized water (diH_2_O) was added to each well. Plates were fixed with 10% formaldehyde in diH_2_O after 48 h. Cells were stained using 1% crystal violet in 20% ethanol and diH_2_O. Viral titers were determined as previously described [21].

### 2.6. Kinase Inhibitor Treatment

Pathway inhibitors were tested for cellular cytotoxicity in Calu-3 cells in order to determine nontoxic testing concentrations. The following inhibitors, acquired from Selleckchem and prepared in DMSO, were tested: GSK690693 (Cat# S1113), SB203580 (Cat# S1076), JNK-IN-8 (Cat# S4901), Dactolisib (BEZ235) (Cat# S1009), PD98059 (Cat# S1177), and LY3214996 (Cat# S8534).

For cellular viability experiments, Calu-3 cells were seeded at 2 × 10^4^ cells/well in 96-well, white-walled plates (Corning, Cat# 3903) and allowed to attach for 24 h. Next, individual inhibitors, over a range of concentrations, were added to the cells. After 24 h of exposure, ATP production was quantified as a measure of cell viability using Cell Titer-Glo assay (Cat# G7570, Promega, Madison, WI, USA). Nontoxic concentrations of inhibitors were identified and utilized in subsequent inhibitor-treatment experiments.

To assess the effects of kinase inhibitors on chemokine/cytokine induction and SARS-CoV-2 infection/replication, Calu-3 cells were seeded into 12-well cell culture plates at 5 × 10^5^ cells/well. Following attachment, cells were pretreated for 1 h with inhibitors. After pretreatment, cells were infected with SARS-CoV-2 (MOI 1) for 1 h. Following initial infection, cells were washed, and were post-treated with the same inhibitor. Cell lysates were collected at 48 hpi and subjected to RNA extraction and RT-qPCR analysis as described above.

### 2.7. Human Specimen Collection and Preparation

A total of 50 human subjects were randomly chosen from patients being tested at the Virginia Tech COVID-19 Molecular Diagnostics Laboratory. All assays are coded and deidentified prior to processing using quantitative real-time PCR to confirm SARS-CoV-2 viral load using testing targeted to the *N*, *E*, and *S* genes and normalized using the human *RPP30* gene. Subject titers ranged from 0 (uninfected controls)—1 × 10^7^ copy numbers. For transcriptomic analysis, RNA specimens were prepared for Clariom^TM^ S Assays following vendor guidelines (ThermoFisher Scientific, Waltham, MA USA). The Clariom S Assay is microarray based and provides extensive coverage of >20,000 well-annotated genes. Briefly, cDNA was generated from 50 ng of pooled RNA, quality and yield were verified following RNase H treatment, and specimens were loaded onto the respective GeneChip Human Transcriptome Array 2.0. Cartridge array hybridization was conducted on the GeneChip^TM^ Instrument, with target hybridization, washing, staining, and scanning. Data was analyzed using the Transcriptome Analysis Console (TAC) 4.0. Further high-resolution pathway analysis was conducted using Ingenuity Pathway Analysis (IPA)^TM^ (Qiagen, Germantown, MD, USA) and CompBio^TM^ (Canopy Bioscience) software.

### 2.8. Statistics

Statistical analyses were calculated using an unpaired, two-tailed Student’s *t*-test or one-way ANOVA with Dunnett’s post-test using Graphpad’s QuickCalcs software. All corresponding adjusted *p*-values are indicated with corresponding significance thresholds within the figure legends. Error is represented as standard error mean (SEM) and replicate numbers are indicated for each respective experiment.

## 3. Results

### 3.1. The Pro-Inflammatory Chemokines CXCL9, CXCL10, and CXCL11 Are Upregulated in Calu-3 Lung Epithelial Cells Following SARS-CoV-2 Infection

**Figure 1:**
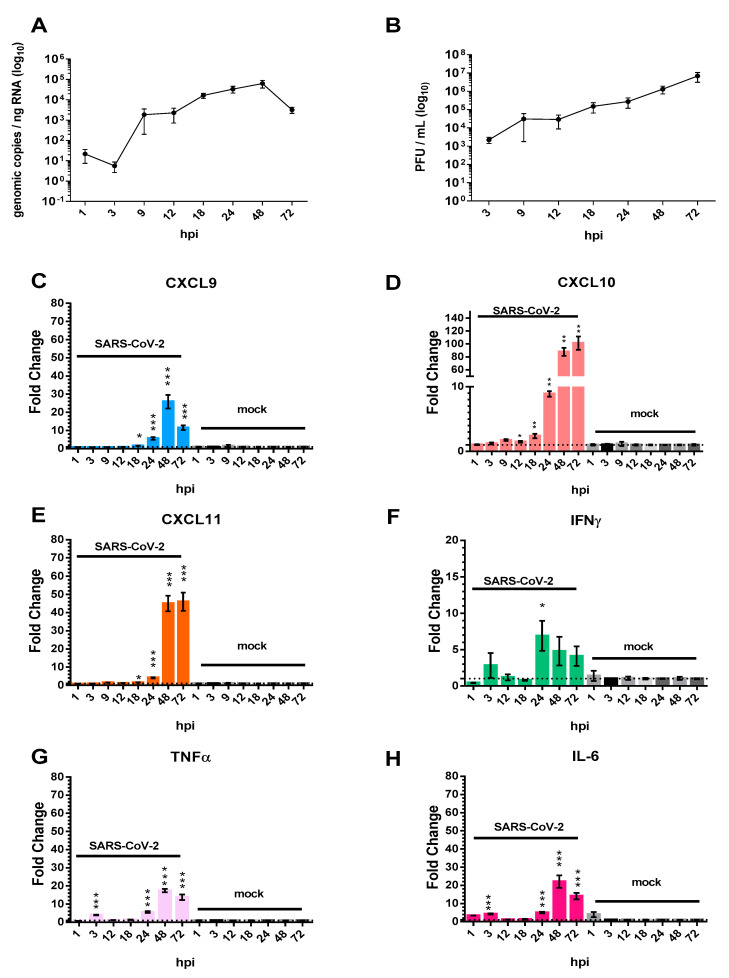
Pro-inflammatory chemokine/cytokine production by Calu-3 cells in response to severe acute respiratory syndrome coronavirus 2 (SARS-CoV-2) infection of Calu-3 cells. Calu-3 cells were infected with SARS-CoV-2 (MOI 1). At 1, 3, 9, 12, 18, 24, 48 and 72 h post-infection (hpi), total cell lysates were collected and subjected to RNA isolation. Supernatants were collected in parallel for viral plaque assays. (**A**) The SARS-CoV-2 viral genome copies detected by RT-qPCR (*n* = 4). (**B**) Viral plaque assays were conducted and PFU/mL calculated (*n* = 4). Host transcriptional-targets were detected by RT-qPCR: (**C**) *CXCL9*, (**D**) *CXCL10*, (**E**) *CXCL11*, (**F**) *IFNγ*, (**G**) *TNFα*, and (**H**) *IL-6*. Transcript levels are expressed as fold change as compared to the respective mock-infected control and represent the mean ± SEM (*n* = 4). Significance was determined using the Student’s *t*-test; * *p* < 0.05, ** *p* < 0.001, *** *p* < 0.0001.

Calu-3 human lung epithelial cells were chosen as a representative in vitro model of SARS-CoV-2 infection to investigate the transcriptional regulation of host chemokines in response to SARS-CoV-2 infection. Indeed, SARS-CoV-2 has been shown previously to infect, and efficiently replicate in, Calu-3 cells [22,23]. Experiments were performed to confirm these results: in an extended time-course experiment, Calu-3 cells were infected at a MOI of 1 for 1 h, washed, and cell lysates/supernatants were collected at 1, 3, 9, 12, 18, 24, 48, and 72 hpi for downstream RT-qPCR and viral plaque assays (Figure 1A,B). At 1 hpi, 2.1 × 10^1^ genomic copies/ng of RNA was observed in cellular lysates prepared from infected Calu-3 cells. By 9 hpi, SARS-CoV-2 genomic copy numbers/ng RNA had increased, a trend that continued over time until the highest genomic RNA quantities were detected at 48 hpi (6.36 × 10^4^ genomic copies/ng RNA). In addition to measuring intracellular replication, viral release kinetics were characterized using viral plaque assays (Figure 1B). At 3 hpi, 2.23 × 10^3^ PFU/mL of SARS-CoV-2 was observed in the supernatants of infected cells. At 12, 18, 24 and 48 hpi, the viral PFU/mL of SARS-CoV-2 increased steadily in increments ranging from a quarter to half a log, reaching 6.87 × 10^6^ PFU/mL at 72 hpi.

The transcriptional expression of *CXCL10*, as well as the related chemokines *CXCL9* and *CXCL11* were assessed by RT-qPCR. Expression of *CXCL9* in infected Calu-3 cells remained low at 1, 3, 9, and 12 hpi (Figure 1C). At approximately 18 hpi there was a 1.6-fold increase in *CXCL9* expression as compared to mock-infected cells. Further increases at 24 hpi (5.7-fold) and 48 hpi (25.9-fold) were observed, prior to moderation by 72 hpi (11.6-fold). Interestingly, other members of the CXC family, *CXCL10* and *CXCL11* displayed similar induction kinetics to that of *CXCL9* but remain elevated at 72 hpi. *CXCL10* expression was found to be significantly increased at 12, 18, 24, 48 and 72 hpi (Figure 1D). The transcriptional expression of *CXCL10* increased from 1.5-fold to 87.7-fold between 12 and 48 hpi. At 72 hpi *CXCL10* transcriptional expression peaked at over 100-fold. *CXCL11* transcriptional expression was statistically increased at 18, 24, 48, and 72 hpi (Figure 1E). *CXCL11* transcriptional expression was the highest 72 hpi with a 46-fold increase.

Host cytokines known to induce the production of CXCL9, CXCL10, and/or CXCL11 include interferon gamma (IFNγ), tumor necrosis factor alpha (TNFα), and interleukin-6 (IL-6) [24,25,26]. To test if these cytokines are induced in response to SARS-CoV-2 infection, and potentially responsible for eliciting the transcriptional induction of chemokine genes, we measured *IFN-γ*, *TNF-α*, and *IL-6* transcript levels over time in infected Calu-3 cells (Figure 1F–H). *IFNγ* gene expression was most significantly upregulated at 24 hpi with a 6.8-fold increase (Figure 1F). At later time-points *IFNγ* expression remained elevated (4.8-fold increase at 48 hpi, and 4.1-fold increase at 72 hpi as compared to mock-infected controls). Of note, in all cases, Ct values for IFNγ were >33, indicating low total levels of this transcript throughout infection. *TNFα* transcript levels were found to be increased at 3, 24, 48 and 72 hpi (Figure 1G). The greatest upregulation of *TNFα* expression was observed at 48 hpi; 17.4-fold increase. *IL-6* gene expression was significantly increased at 24 hpi (5-fold), continued to increase at 48 hpi (22-fold), and then moderated at 72 hpi (14-fold).

### 3.2. Chemokine/Cytokine Induction in the Lungs of SARS-CoV-2-Infected hACE2 Mice

To characterize the induction of pro-inflammatory chemokines in vivo following SARS-CoV-2 infection, K18-hACE2 transgenic mice were utilized [27]. The K18-hACE2 model was developed by using a K18 promotor to express hACE2 (the major cellular receptor for viral entry) in airway epithelial cells of C57BL/6 mice [28]. Infection with SARS-CoV-2 in this humanized mouse strain results in high viral titers in the lung that correlate with significant lung inflammation and a progressive decline in lung function [29]. In the current study, female hACE2 expressing mice were challenged intranasally with 1 × 10^5^ PFU of SARS-CoV-2 and either followed for survival or serial sacrificed at 1, 2, 3, 4 and 5 dpi for organ harvest and endpoint analyses. Uninfected hACE2 mice (labeled as day 0) were utilized as controls.

**Figure 2:**
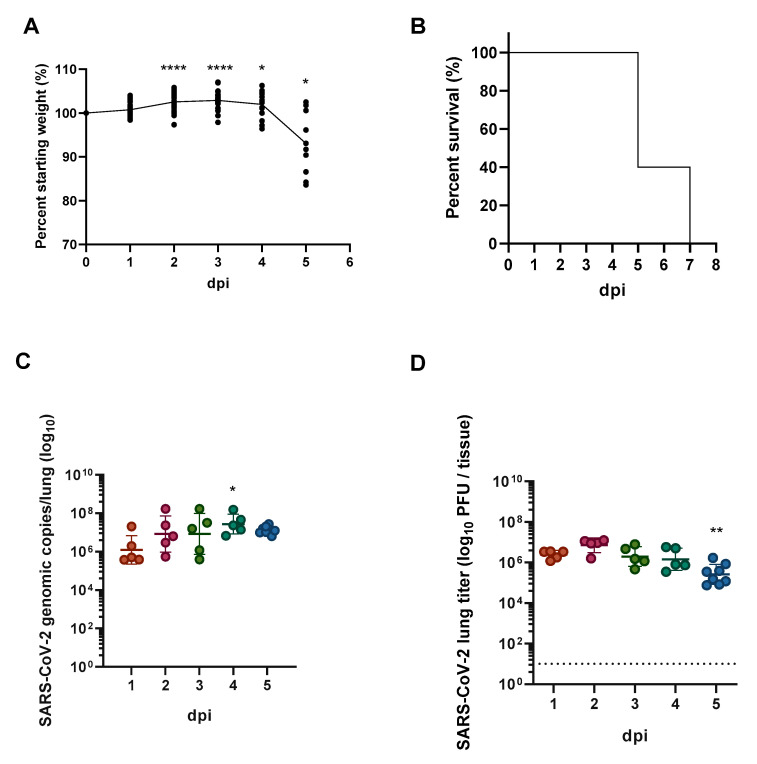
SARS-CoV-2 causes fatal disease in hACE2 mice and replicates in the lungs. Female hACE2 mice were intranasally exposed to 1 × 10^5^ PFU of SARS-CoV-2. Mice were followed for survival and sacrificed at 1, 2, 3, 4 and 5 dpi. Lung homogenates were prepared from infected mice for RNA isolation and RT-qPCR. (**A**) Percent of starting weight over time for all infected mice. (**B**) Kaplan–Meier survival curve of hACE mice infected with SARS-CoV-2 (*n* = 5). (**C**) SARS-CoV-2 viral genomic copies measured over time within the lungs by RT-qPCR. Mean copy number (log10) ± SEM is shown (*n* = 5 per time point, except 5 dpi *n* = 8). (**D**) SARS-CoV-2 viral titer within the lungs as determined via plaque assays. Mean titer (log10) ± SEM is shown (*n* = 5 per time point, except 5 dpi *n* = 8). Statistical significance was determined using one-way ANOVA with Dunnett’s post-hoc analysis with significance relevant to 0 dpi (panel **A**) or 1 dpi (panels **C** and **D**). * *p* < 0.05, ** *p* < 0.01, and **** *p* < 0.0001.

SARS-CoV-2-infected mice progressively lost weight during the course of infection (Figure 2A). Of the five mice in the survival group, three animals were euthanized at 5 dpi due to weight loss (>15%) and clinical symptoms; the remaining two mice were euthanized at 7 dpi (Figure 2B). Thus, there was 100% mortality in this model. Viral burden was measured in the lungs of animals by RT-qPCR and plaque assays. Extracted RNA from mice at each time-point was subjected to RT-qPCR for detection of the *N* gene of SARS-CoV-2 (Figure 2C). Viral RNA was detected in the lungs of infected mice at 1 dpi with a mean SARS-CoV-2 genomic copies/lung being 2.36 × 10^7^. Subsequent replication and sustained infection were observed throughout analysis with a range of 1–2 × 10^8^ genomic copies/lung tissue. Additionally, viral replication kinetics measured by viral plaque assays using whole-lung tissue homogenates showed that 1.5 × 10^6^ PFU/mL was detected in lung tissue at 1 dpi (Figure 2D). At 2 dpi peak viral titer was measured in lungs of infected mice (7.94 × 10^6^ PFU/mL). At later time points, viral titers declined, reaching 2.51 × 10^5^ PFU/mL at 5 dpi. Collectively, these findings demonstrate the sustainability of SARS-CoV-2 infection and replication in the lungs of hACE2 mice. 

**Figure 3:**
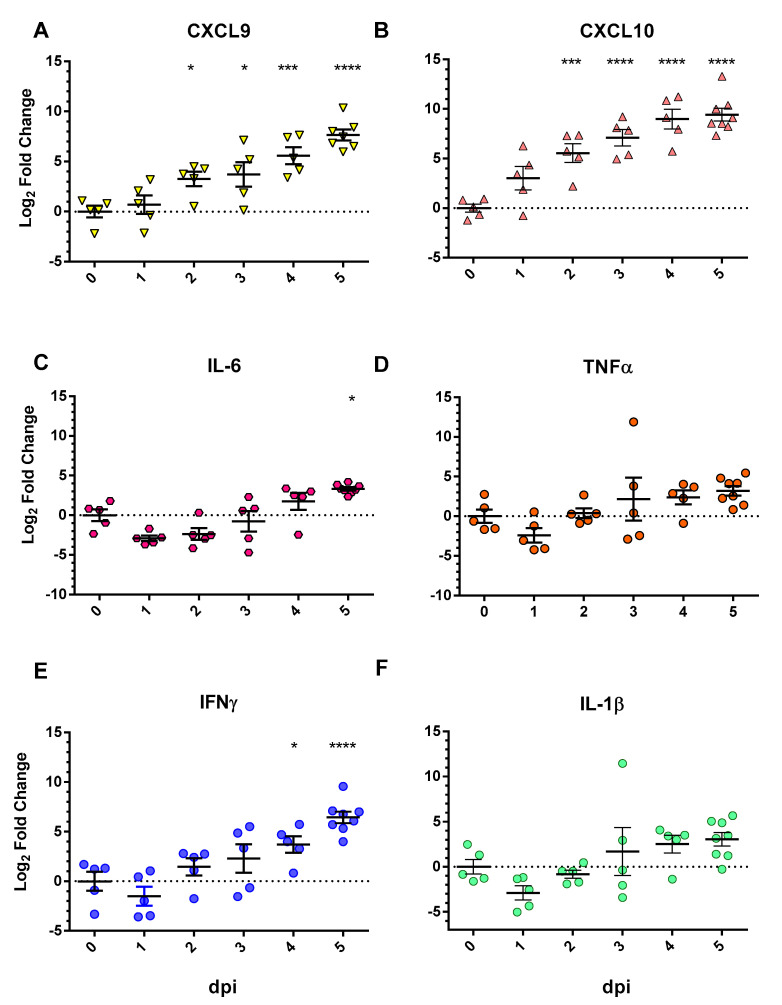
Pro-inflammatory chemokine/cytokine induction in the lungs of hACE2 mice following SARS-CoV-2 infection. hACE2 mice were challenged intranasally with 1×10^5^ PFU of SARS-CoV-2 and serial sacrificed at 1, 2, 3, 4, 5 dpi. Following sacrifice, lung tissue was harvested for RNA isolation and RT-qPCR analysis (*n* = 5 per time point, except 5 dpi *n* = 8). Pro-inflammatory chemokines and cytokines were measured: (**A**) *CXCL9*, (**B**) *CXCL10*, (**C**) *IFNγ*, (**E**) *TNFα*, (**D**) *IL-6*, and (**F**) *IL-1β*. Transcript levels are expressed as Log2 fold change compared to uninfected (0 dpi) controls and represent the mean ± SEM. Statistical significance was determined using one-way ANOVA with Dunnett’s post-hoc analysis. Corresponding significance levels represent statistically significant differences between each time-point as compared to 0 dpi and are indicated respectively: * *p* < 0.05, *** *p* < 0.001, and **** *p* < 0.0001.

To assess the expression of pro-inflammatory chemokine and cytokine genes in SARS-CoV-2-infected mice, transcript levels for *CXCL9*, *CXCL10*, *IL-6*, *TNFα*, *IFNγ*, and *IL-1β* were measured from the lungs of serially sacrificed animals by RT-qPCR. *CXCL11* gene expression was not assessed due to it not being expressed in C57BL/6 mice [30]. *CXCL9* and *CXCL10* mRNA levels were significantly elevated at 2–5 dpi (Figure 3A,B). While there is variability in *CXCL9* and *CXCL10* gene expression among animals, there is an evident trend towards upregulation of transcripts over time with large increases in transcript level elevation at days 2–5 with increasing statistical significance when compared to uninfected (0 dpi). *IL-6* levels were significantly increased at 5 dpi with overall time-dependent elevation in IL-6 transcripts (Figure 3E); *TNFα* levels, while not statistically significant, showed a trend of upregulation over time (Figure 3D). It is also interesting to note the magnitude of the changes, with *CXCL10* levels being on average 5–10× higher than *CXCL9* and *IL-6* levels and over 50X higher than *TNFα* mRNA levels. *IFNγ* transcript levels were trending upward over time with statistically significant increases observed from 4–5 dpi (Figure 3E). The expression of *IL-1β*, another inducer of *CXCL10* gene transcription, was similarly lower in magnitude as compared to the CXC’s, and while transcript levels increased over time, was not deemed to be statistically significant (Figure 3F) [31].

### 3.3. AKT Inhibitor, GSK690693, Prevents the Induction of CXCL9, CXCL10 and CXCL11 by SARS-CoV-2 in Calu-3 Cells

**Figure 4:**
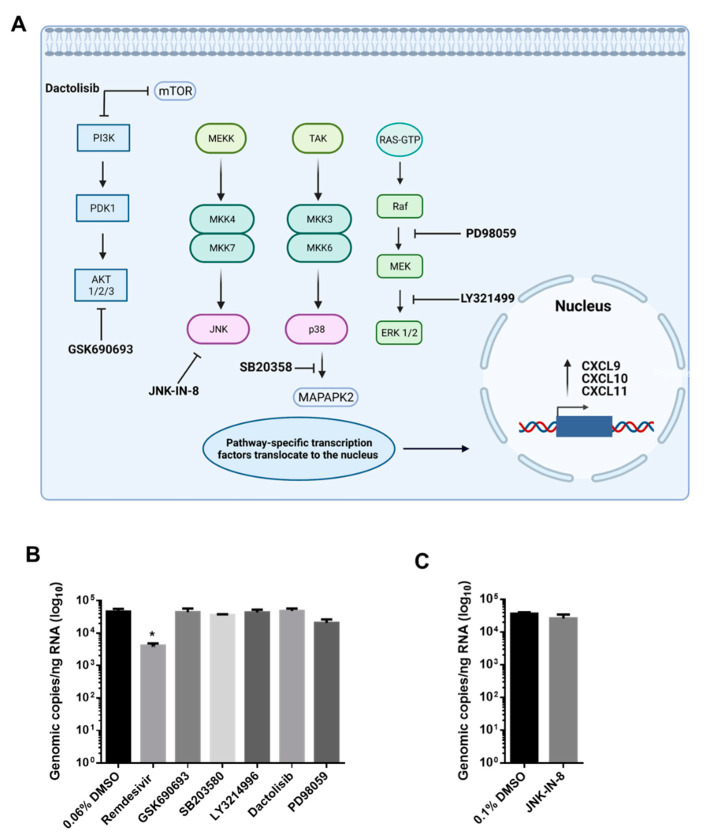
Kinase inhibitors targeting intracellular signaling-pathways that regulate *CXCL9*, *CXCL10*, and/or *CXCL11* transcription do not affect SARS-CoV-2 RNA production. Created with BioRender.com. (**A**) Inhibitors of the pathways referenced, PI3K/AKT, JNK, p38 MAPK, and ERK/MEK were utilized to study chemokine transcriptional modulation. (**B**) Calu-3 cells were pretreated for 1 h with the indicated kinase inhibitor at nontoxic concentrations: Remdesivir (1 µM) control, GSK690693 (50 µM), SB203580 (25 µM), Dactolisib (10 nM), LY3214996 (100 nM), PD98059 (100 µM), and (**C**) JNK-IN-8 (5 µM). Cells were then infected with SARS-CoV-2 (MOI 1) and post-treated with the same inhibitor. Cell lysates were collected at 48 hpi and subjected to RNA isolation and RT-qPCR for the detection of SARS-CoV-2 intracellular RNA levels. Mean copy number (log10) ± SEM is shown (*n* = 3 per time point). Significance was determined using the Student’s *t*-test; * *p* < 0.05.

Activation of multiple signal transduction pathways are known to stimulate *CXCL10* transcription including AKT, p38 MAPK, PI3K, ERK, and JNK signaling pathways [32,33]. Additionally, the induction of these signaling pathways has been associated with *CXCL9* and *CXCL11* production in various cell types [34]. To determine which pathway(s) is contributing to the induction of these CXC chemokines upon SARS-CoV-2 infection, small molecule inhibitors of AKT (GSK690693), p38 MAPK (SB203580), PI3K (Dactolisib), MEK (PD98059), ERK (LY3214996), and JNK (JNK-IN-8) were employed. The specific targets of the small molecule inhibitors and locational impact on the referenced pathways is displayed in Figure 4A. Cellular viability assays using Calu-3 cells were first used to identify nontoxic concentrations of the above kinase inhibitors (Supplemental Figure S1). Minimal cytotoxic effects were observed at inhibitor concentrations previously shown to inhibit target kinases.

Based on these results, the following inhibitor concentrations were selected for further testing: GSK690693 (50 µM), SB203580 (25 µM), Dactolisib (10 nM), LY3214996 (100 nM), PD98059 (100 µM), and JNK-IN-8 (5 µM). Calu-3 cells were treated with nontoxic concentrations of inhibitors for 1 h prior to SARS-CoV-2 infection. Cells were then infected with SARS-CoV-2 (MOI 1) and post-treated with the same inhibitor. RNA samples were collected at 48 hpi for quantifying *CXCL9*, *CXCL10*, *CXCL11* gene expression and viral RNA levels. Remdesivir (1 µM), a known SARS-CoV-2 antiviral, was utilized as a control for antiviral activity [35]. Potential effects of kinase inhibitors on SARS-CoV-2 infection/replication inhibitors were evaluated. None of the examined inhibitors had a significant effect on SARS-CoV-2 intracellular RNA levels when measured by RT-qPCR (Figure 4B,C). Treatment of Calu-3 cells with a low dose of Remdesivir (1 µM) resulted in a significant reduction in SARS-CoV-2 viral RNA with an approximate 1.12-log_10_ reduction in genomic copies/ng RNA (Figure 4B). Accordingly, the potential impact of the inhibitors on chemokine induction is not due to a decrease in viral replication.

**Figure 5:**
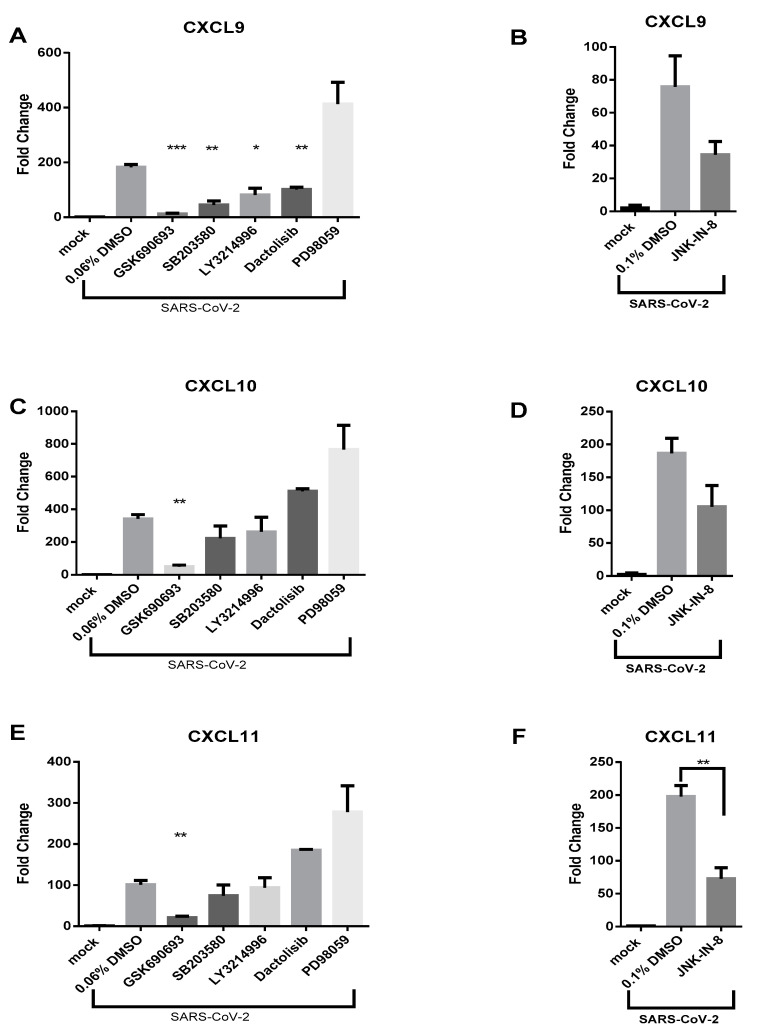
The inhibition of AKT prevents CXCL9, CXCL10, and CXCL11 chemokine induction by SARS-CoV-2. RNA samples generated as described in Figure 4 were subjected to RT-qPCR analysis of *CXCL9* (**A**,**B**), *CXCL10* (**C**,**D**), and *CXCL11* (**E**,**F**) gene expression. Transcript levels are expressed as fold change compared to infected cells exposed to vehicle alone and represent the mean ± SEM (*n* = 3). Significance was determined using the Student’s *t*-test; * *p* < 0.05, ** *p* < 0.001, *** *p* < 0.0001.

GSK690693-treated cells displayed a near complete absence of *CXCL9* induction as compared to SARS-CoV-2-infected Calu-3 cells treated with vehicle alone (Figure 5A). Specifically, treatment with GSK690693 resulted in 14.85-fold less transcriptional upregulation of *CXCL9* expression and treatment with SB203580 resulted in 4.08-fold less *CXCL9* transcripts. LY3214996- and Dactolisib-treated cells displayed less pronounced reductions in *CXCL9* induction, while PD98059 and JNK-IN-8 did not statistically reduce *CXCL9* transcript levels following infection (Figure 5A,B). Of all the kinase inhibitors tested, only the AKT inhibitor GSK690693 significantly reduced *CXCL10* transcription (Figure 5C,D). *CXCL10* transcription was 6.93-fold less as compared to infected cells exposed to vehicle alone in the presence of GSK690693 (Figure 5C). Regarding *CXCL11*, GSK690693 (4.82-fold reduction) and JNK-IN-8 (2.72-fold reduction) each significantly reduced *CXCL11* induction in response to infection as compared to infected cells treated with vehicle alone (Figure 5E,F). Overall, the induction of *CXCL9*, *CXCL10* and *CXCL11* transcription was most significantly precluded by inhibition of AKT and this impact was not due to reduction of viral replication.

### 3.4. The CXCL9/10/11 Axis Is Significantly Upregulated in SARS-CoV-2 Positive Human Subjects

**Figure 6:**
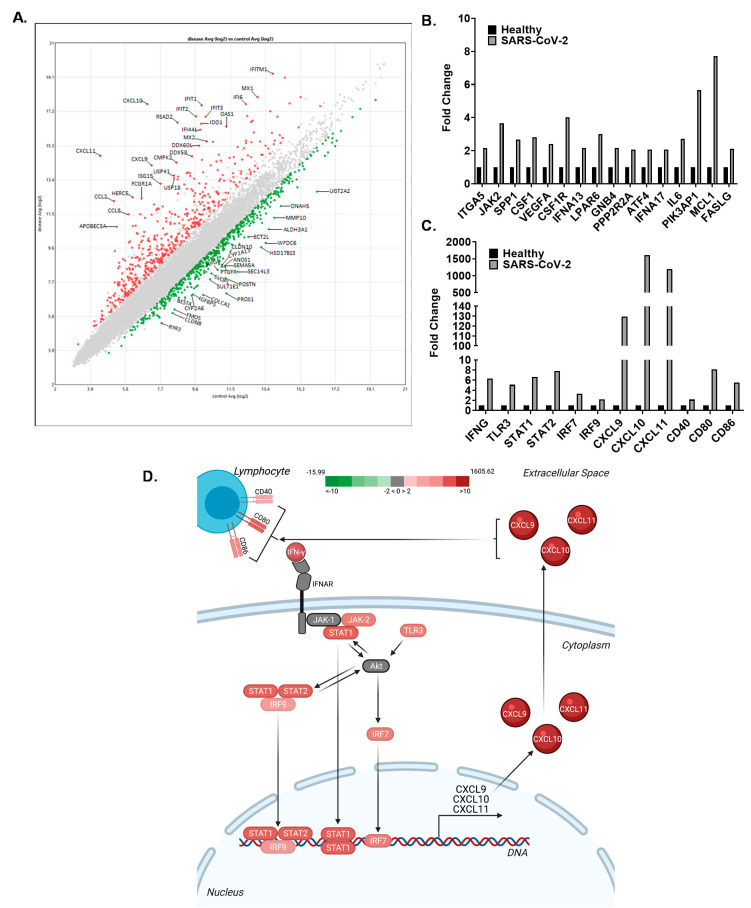
*CXCL9*, *CXCL10*, and *CXCL11* are significantly upregulated in human subjects following SARS-CoV-2 infection. Gene expression was profiled using Clariom S Assays and RNA collected from human subjects being screened for SARS-CoV-2. Pathway analysis was conducted using the Transcriptome Analysis Console, Ingenuity Pathway Analysis, and CompBio software. (**A**) Scatter plots were generated based on fold-change with the top 25 upregulated (red) and downregulated (green) genes labeled on the graph. (**B**) Pathway analysis indicated that AKT formed a critical signaling hub that was upregulated following virus infections, and fold change for the components associated with this signaling were graphed. (**C**) *CXCL9*, *CXCL10*, and *CXCL11* were significantly upregulated in infected subjects, with the fold change of key signaling components graphed. (**D**) Schematic representation of the pathway analysis data, with heat map superimposed, defining the key components associated with the increased *CXCL9*, *CXCL10*, and *CXCL11* generation and biological functions after SARS-CoV-2 infection. *n* = 35 SARS-CoV-2 positive subjects; 15 SARS-CoV-2 negative subjects (controls).

Transcriptomic analysis comparing human subjects that were positive for SARS-CoV-2 with subjects that had no detectable levels of the virus (controls) at the time of testing revealed significant changes in a wide range of genes. In general, the findings were consistent with other similar studies already published, including an inverse relationship between type-I interferon production (decreased) and cytokine/chemokine signaling (increased) [36]. A scatterplot analysis of the individual genes found to be significantly upregulated or downregulated in the SARS-CoV-2 subjects compared to the controls was generated and analyzed (Figure 6A). The top 25 genes upregulated (red) and downregulated (green) are labeled (Figure 6A). Consistent with the data discussed throughout this manuscript, the largest increases were observed in *CXCL10* (1606 fold increase), *CXCL11* (1192 fold increase), and *CXCL9* (129 fold increase) in the SARS-CoV-2 infected individuals (Figure 6A,C). Pathway analysis assessments of the transcriptomics data revealed a significant increase in AKT signaling, associated with the significant upregulation of 16 key components of this signaling pathway (Figure 6B). While *AKT* itself was not significantly altered following SARS-CoV-2 infection, significant changes in these pathway components indicate that signaling associated with AKT is significantly increased. Pathway analysis further predicted that the increase in *CXCL9*, *CXCL10*, and *CXCL11* in the human subjects was associated with increased IFNγ and TLR3 signaling (Figure 6C,D). Downstream from these signaling hubs, *STAT1*, *STAT2*, *IRF9*, and *IRF7* are significantly upregulated and were identified as key transcription factors associated with *CXCL9*, *CXCL10*, and *CXCL11* following SARS-CoV-2 infection (Figure 6C,D). Pathway analysis further suggests that the increased chemokine signaling is associated with increased CD40, CD80, and CD86 signaling that was also upregulated in the human subjects following SARS-CoV-2 (Figure 6C,D).

## 4. Discussion

The induction of *CXCL10* is a key marker of nonviral and viral ARDS and findings presented herein show that in response to SARS-CoV-2 infection, *CXCL10*, and the related chemokines *CXCL9* and *CXCL11*, are strongly induced in human lung epithelial cells, in a murine infection model, and in humans infected with SARS-CoV-2. *CXCL9*, *CXCL10*, and *CXCL11* are a subfamily of immunostimulatory CXC-chemokines that act in both autocrine and paracrine fashion through their shared cellular receptor CXCR3 that is expressed on diverse cell types such as epithelial cells, endothelial cells, B and T lymphocytes, macrophages, natural killer cells and dendritic cells [33,37,38,39]. The current study profiles *CXCL9, CXL10,* and *CXCL11* transcriptional expression during SARS-CoV-2 infection of Calu-3 cells, in SARS-CoV-2 infected hACE2 mice, and in SARS-CoV-2 patient samples. It is the first to show that transcription of all three of these chemokines is dependent on AKT signaling in SARS-CoV-2 infected cells.

*CXCL10* and highly homologous associated members, *CXCL9* and *CXCL11*, are immune chemokines that are members of the CXC-family of chemokines and are often referred to as IFN-inducible CXCR3 chemokines [37]. These three chemokines share IFNγ as a primary inducer and CXCR3 as a primary G protein coupled receptor (GPCR). Interestingly, *IFNγ*, a major inducer of *CXCL10*, was upregulated following infection of Calu-3 cells, but was still at relatively low levels (Figure 1F). In hACE2 SARS-CoV-2 infected mice, it was significantly upregulated at 4 and 5 dpi (Figure 3E). The delayed upregulation of IFNγ observed in these studies is consistent with the findings of other groups and suggests that IFNγ is not the only stimulator of *CXCL10* family members during SARS-CoV-2 infection [36,40,41]. Therefore, other cytokines impacting CXC transcription were explored. IL-1β has been shown to be an activator of *CXCL10* transcription and synergistic to IFNγ-induced production of *CXCL10* [31]. However, over the course of SARS-CoV-2 infection in mice, *IL-1β* transcription was not significantly elevated (Figure 3F). *TNFα*, a known inducer and antagonist of *CXCL10* expression, was highly upregulated in SARS-CoV-2 infected Calu-3 cells at 3 hpi and consistently from 24 to 72 hpi (Figure 1G). However, *TNFα* was not significantly upregulated in the lungs of SARS-CoV-2 infected mice (Figure 3D). Lastly, *IL-6* transcriptional levels displayed slight upregulation of transcripts at 3 hpi and then significant increases from 24 to 48 hpi in Calu-3 cells (Figure 1H). *IL-6* was significantly upregulated at 5 dpi in the SARS-CoV-2 infected hACE2 mice (Figure 3C). In summary, a consistent pattern of upregulation of pro-inflammatory cytokines that are also inducers of the investigated CXC chemokines was observed in vitro; however, these results were not completely recapitulated in the lungs of SARS-CoV-2 infected hACE2 mice. Lung samples were processed as homogenized samples and thus contain a mixture of cell types which may contribute to the differences observed in vitro and in vivo.

Interestingly, IL-1β in addition to being involved in *CXCL10* regulation is a major inducer of TNFα and IL-6 secretion from lung macrophages and mast cells following pathogen stimulation [42,43]. However, in reference to the hACE2 studies conducted in this paper, *IL-1β* and *TNFα* transcriptional expression, while increasing over time, are not significantly upregulated at any time-point in the lungs of mice. Other groups have also observed lack of IL-1β cytokine production in the lung tissue of SARS-CoV-2 infected hACE2 mice, but observed increases in brain tissue [44]. In human serum samples, IL-1β activation is robust and correlated with severe disease pathologies and is thought to in turn activate TNFα and IL-6 production [45,46,47,48]. Interestingly, IL-1β along with pathogenic microorganisms are capable of activating toll like receptors (TLRs) and through synergistic activation of these two cytokine-stimulating mechanisms may contribute to a cytokine storm via cytokine overproduction [42,43]. The coproduction of these inflammatory mediators has led to investigation of IL-1 agonists for combatting the cytokine storm and severe pathogenic symptoms in SARS-CoV-2 infected patients [42,49]. Utilization of IL-1 receptor agonists could additionally inhibit induction of downstream targets of IL-1β, such as TNFα, IL-6 and CXCL10 thus mediating the overall inflammatory state induced by SARS-CoV-2.

In prior studies by Wong et al. conducted with SARS-CoV, patients with severe pulmonary manifestations were observed to have elevated serum levels of pro-inflammatory chemokines and cytokines, such as CXCL10, IL-6, IL-8, and IFN-γ [13]; these findings were confirmed in human clinical samples. Investigation of SARS-CoV infection in BALB/c mice demonstrated a biphasic pro-inflammatory state that correlated with an early and late immune response [50]. In this non-lethal model, infected BALB/c mice showed elevated levels of pro-inflammatory cytokines (RNA and protein), and specifically highly-induced levels of CXCL10 at both 2 and 7 dpi; correlating to the early and late immune response [50]. The biphasic expression of CXCL10 correlated with viral titer at 2 dpi, but not at 7 dpi, suggesting viral replication and chemokine secretion are not necessarily simultaneous or in parallel. Importantly, the late induction of CXCL10 was associated with immune cell infiltration, pneumonitis, and alveolar damage [50]. CXCL9 was also found to be induced late in the course of disease. Our findings are consistent with other studies reporting elevated serum CXCL10 levels as well as transcriptional expression in PBMC and BALF in SARS-CoV-2 infected patients [15,17]. High levels of CXCL10 have also been observed to be expressed during SARS-CoV-2 infection and are associated with lung injury in SARS-CoV-2 patients [18]. Ultimately, the substantial induction of pro-inflammatory CXC-chemokine ligands may contribute to the development of viral ARDS in the context of SARS-CoV-2.

In order to better understand which signaling pathways are most significantly contributing to the robust chemokine induction and subsequent hyper-inflammatory response observed during SARS-CoV-2 infections, selective inhibitors targeting pathways responsible for inducing *CXCL9*, *CXCL10* and *CXCL11* transcription were utilized. In the current study, small molecule inhibitors of four major pathways (PI3K/AKT, p38 MAPK, JNK, and ERK/MEK) that contribute to *CXCL10* transcription were targeted (Figure 4A). It was found that inhibiting these pathways did not result in an antiviral effect or reduction in intracellular viral RNA (Figure 4B,C). However, the AKT (GSK690693) inhibitor prevented the induction of *CXCL9*, *CXCL10*, and *CXCL11* in Calu-3 cells following SARS-CoV-2 infection (Figure 5), indicating that this is the primary pathway needed to stimulate gene expression on these chemokines in SARS-CoV-2 infected cells. Pathway analysis of transcriptomic data from SARS-CoV-2 infected patients was consistent with these results, showing that 16 components of the AKT signaling pathway were upregulated.

GSK690693 is a pan-AKT inhibitor, targeting AKT1, AKT2 and AKT3 which is downstream of the P13K signaling cascade (Figure 4A). Interestingly, Dactolisib, a PI3K and mTOR inhibitor, had no impact on *CXCL10* and *CXCL11* transcription, but a greater magnitude and significant reduction in *CXCL9* transcription. These results indicate that transcription of the investigated CXC chemokines are differentially regulated following SARS-CoV-2 infection. They also suggest that AKT can regulate *CXCL9* transcription independent of PI3K. In fact, AKT can be activated through non-canonical pathways independent of PI3K; Ack1, TNK and Src are some known non-canonical activators of AKT shown to function even under PI3K inhibition [51,52,53,54,55]. Perhaps during SARS-CoV-2 infection of Calu-3, inhibition of PI3K is not sufficient for substantially reducing the induction of chemokine expression because of non-canonical AKT activation. Interestingly, P13K-AKT signaling has been shown to play a regulatory role in several models of lung injury. Studies conducted on key regulatory genes contributing to ALI, have shown that P13K-AKT signaling (and inhibition of) reduces gene expression associated with pro-hypoxemia in lung epithelial cells [56]. Additionally, in the context of a ventilator-induced lung injury, the inhibition of P13K-AKT signaling pathway results in reduced ventilator associated injury [57].

This study provides further characterization of the SARS-CoV-2 mediated inflammatory state by characterizing chemokine expression and delineating specific arms of cellular signaling pathways that potentiate inflammation in response to SARS-CoV-2 infection. Utilizing pathway-specific immunomodulatory inhibitors for reducing the production of CXC chemokines, in conjunction with antiviral therapies, could be particularly useful for the treatment and prevention of severe SARS-CoV-2 associated pathologies such as ALI or ARDS. An example of a successful combinatorial antiviral therapy and immunomodulatory approach is Remdesivir and Barcitinib (JAK-1 inhibitor), which has been shown to be effective in reducing clinical severity and mortality rates in COVID-19 patients [58]. Miltefosine is an FDA approved drug that inhibits PI3K/AKT and is used for the treatment of leishmaniasis [59,60]. Based on the work presented within, future studies should explore the combination of Remdesivir and AKT inhibitors, such as Miltefosine, for the treatment of COVID-19.

## Data Availability

Data is contained within the article and supplementary material.

## Supplementary Materials

The following are available online at https://www.mdpi.com/article/10.3390/v13061062/s1, Figure S1: Cytotoxicity of kinase inhibitors in Calu-3 cells.

## References

[1] Jin Y., Yang H., Ji W., Wu W., Chen S., Zhang W., Duan G. (2020). Virology, Epidemiology, Pathogenesis, and Control of COVID-19. Viruses.

[2] Zheng J. (2020). SARS-CoV-2: An Emerging Coronavirus that Causes a Global Threat. Int. J. Biol. Sci..

[3] Zhou P., Yang X.-L., Wang X.-G., Hu B., Zhang L., Zhang W., Si H.-R., Zhu Y., Li B., Huang C.-L. (2020). A pneumonia outbreak associated with a new coronavirus of probable bat origin. Nature.

[4] Harrison A.G., Lin T., Wang P. (2020). Mechanisms of SARS-CoV-2 Transmission and Pathogenesis. Trends Immunol..

[5] Martines R.B. (2020). Pathology and Pathogenesis of SARS-CoV-2 Associated with Fatal Coronavirus Disease, United States. Emerg. Infect. Dis..

[6] Dhama K. (2020). Coronavirus Disease 2019-COVID-19. Clin. Microbiol. Rev..

[7] Khalaf K. (2020). SARS-CoV-2: Pathogenesis, and Advancements in Diagnostics and Treatment. Front. Immunol..

[8] Wu D., Wu T., Liu Q., Yang Z. (2020). The SARS-CoV-2 outbreak: What we know. Int. J. Infect. Dis..

[9] Felsenstein S., Herbert J.A., McNamara P.S., Hedrich C.M. (2020). COVID-19: Immunology and treatment options. Clin. Immunol..

[10] Dhama K. (2020). An update on SARS-CoV-2/COVID-19 with particular reference to its clinical pathology, pathogenesis, immunopathology and mitigation strategies. Travel Med. Infect. Dis..

[11] Chen N. (2020). Epidemiological and clinical characteristics of 99 cases of 2019 novel coronavirus pneumonia in Wuhan, China: A descriptive study. Lancet.

[12] Ragab D., Eldin H.S., Taeimah M., Khattab R., Salem R. (2020). The COVID-19 Cytokine Storm; What We Know So Far. Front. Immunol..

[13] Wong C.K., Lam C.W.K., Wu A.K.L., Ip W.K., Lee N.L.S., Chan I.H.S., Lit L.C.W., Hui D.S.C., Chan M.H.M., Chung S.S.C. (2004). Plasma inflammatory cytokines and chemokines in severe acute respiratory syndrome. Clin. Exp. Immunol..

[14] Mahallawi W.H., Khabour O.F., Zhang Q., Makhdoum H.M., Suliman B.A. (2018). MERS-CoV infection in humans is associated with a pro-inflammatory Th1 and Th17 cytokine profile. Cytokine.

[15] Huang C. (2020). Clinical features of patients infected with 2019 novel coronavirus in Wuhan, China. Lancet.

[16] Liang Y., Wang M.-L., Chien C.-S., Yarmishyn A.A., Yang Y.-P., Lai W.-Y., Luo Y.-H., Lin Y.-T., Chen Y.-J., Chang P.-C. (2020). Highlight of Immune Pathogenic Response and Hematopathologic Effect in SARS-CoV, MERS-CoV, and SARS-Cov-2 Infection. Front. Immunol..

[17] Xiong Y., Liu Y., Cao L., Wang D., Guo M., Jiang A., Guo D., Hu W., Yang J., Tang Z. (2020). Transcriptomic characteristics of bronchoalveolar lavage fluid and peripheral blood mononuclear cells in COVID-19 patients. Emerg. Microbes Infect..

[18] Ichikawa A., Kuba K., Morita M., Chida S., Tezuka H., Hara H., Sasaki T., Ohteki T., Ranieri V.M., Dos Santos C.C. (2013). CXCL10-CXCR3 Enhances the Development of Neutrophil-mediated Fulminant Lung Injury of Viral and Nonviral Origin. Am. J. Respir. Crit. Care Med..

[19] Coperchini F., Chiovato L., Ricci G., Croce L., Magri F., Rotondi M. (2021). The cytokine storm in COVID-19: Further advances in our understanding the role of specific chemokines involved. Cytokine Growth Factor Rev..

[20] Coperchini F., Chiovato L., Rotondi M. (2021). Interleukin-6, CXCL10 and Infiltrating Macrophages in COVID-19-Related Cytokine Storm: Not One for All But All for One!. Front. Immunol..

[21] Baer A., Kehn-Hall K. (2014). Viral Concentration Determination Through Plaque Assays: Using Traditional and Novel Overlay Systems. J. Vis. Exp..

[22] Chu H., Chan J., Yuen T., Shuai H., Yip C., Tsang J., Huang X., Chai Y., Yang D., Hou Y. (2020). Comparative tropism, replication kinetics, and cell damage profiling of SARS-CoV-2 and SARS-CoV with implications for clinical manifestations, transmissibility, and laboratory studies of COVID-19: An observational study. Lancet Microbe.

[23] Bestle D. (2020). TMPRSS2 and furin are both essential for proteolytic activation of SARS-CoV-2 in human airway cells. Life Sci. Alliance.

[24] Tokunaga R., Zhang W., Naseem M., Puccini A., Berger M.D., Soni S., McSkane M., Baba H., Lenz H.-J. (2018). CXCL9, CXCL10, CXCL11/CXCR3 axis for immune activation—A target for novel cancer therapy. Cancer Treat. Rev..

[25] Alomar S.Y. (2016). IL-1beta (interleukin-1beta) stimulates the production and release of multiple cytokines and chemokines by human preadipocytes. Arch. Physiol. Biochem..

[26] McLoughlin R., Jenkins B.J., Grail D., Williams A.S., Fielding C., Parker C.R., Ernst M., Topley N., Jones S.A. (2005). IL-6 trans-signaling via STAT3 directs T cell infiltration in acute inflammation. Proc. Natl. Acad. Sci. USA.

[27] Hoffmann M. (2020). SARS-CoV-2 Cell Entry Depends on ACE2 and TMPRSS2 and Is Blocked by a Clinically Proven Protease Inhibitor. Cell.

[28] McCray P.B., Pewe L., Wohlford-Lenane C., Hickey M., Manzel L., Shi L., Netland J., Jia H.P., Halabi C., Sigmund C.D. (2007). Lethal Infection of K18-hACE2 Mice Infected with Severe Acute Respiratory Syndrome Coronavirus. J. Virol..

[29] Winkler E.S. (2020). SARS-CoV-2 infection of human ACE2-transgenic mice causes severe lung inflammation and impaired function. Nat. Immunol..

[30] Qi Z., Wang J., Han X., Yang J., Zhao G., Cao Y. (2014). Listr1 locus regulates innate immunity against Listeria monocytogenes infection in the mouse liver possibly through Cxcl11 polymorphism. Immunogenetics.

[31] Burke S.J. (2013). Synergistic expression of the CXCL10 gene in response to IL-1beta and IFN-gamma involves NF-kappaB, phosphorylation of STAT1 at Tyr701, and acetylation of histones H3 and H4. J. Immunol..

[32] Vazirinejad R., Ahmadi Z., Arababadi M.K., Hassanshahi G., Kennedy H. (2014). The Biological Functions, Structure and Sources of CXCL10 and Its Outstanding Part in the Pathophysiology of Multiple Sclerosis. Neuroimmunomodulation.

[33] Liu M., Guo S., Hibbert J.M., Jain V., Singh N., Wilson N.O., Stiles J.K. (2011). CXCL10/IP-10 in infectious diseases pathogenesis and potential therapeutic implications. Cytokine Growth Factor Rev..

[34] Fenwick P.S., Macedo P., Kilty I.C., Barnes P.J., Donnelly L.E. (2015). Effect of JAK Inhibitors on Release of CXCL9, CXCL10 and CXCL11 from Human Airway Epithelial Cells. PLoS ONE.

[35] Choy K.T. (2020). Remdesivir, lopinavir, emetine, and homoharringtonine inhibit SARS-CoV-2 replication in vitro. Antivir. Res..

[36] Blanco-Melo D. (2020). Imbalanced Host Response to SARS-CoV-2 Drives Development of COVID-19. Cell.

[37] Metzemaekers M., Vanheule V., Janssens R., Struyf S., Proost P. (2018). Overview of the Mechanisms that May Contribute to the Non-Redundant Activities of Interferon-Inducible CXC Chemokine Receptor 3 Ligands. Front. Immunol..

[38] Woodland D.L., Scott I. (2005). T Cell Memory in the Lung Airways. Proc. Am. Thorac. Soc..

[39] (1971). Edward Arthur Steinhaus, 1914–1969. Annu. Rev. Entomol..

[40] Chu H. (2020). Comparative Replication and Immune Activation Profiles of SARS-CoV-2 and SARS-CoV in Human Lungs: An Ex Vivo Study With Implications for the Pathogenesis of COVID-19. Clin. Infect. Dis..

[41] Yang D. (2020). Attenuated Interferon and Proinflammatory Response in SARS-CoV-2-Infected Human Dendritic Cells Is Associated With Viral Antagonism of STAT1 Phosphorylation. J. Infect. Dis..

[42] Conti P. (2020). Coronavirus-19 (SARS-CoV-2) induces acute severe lung inflammation via IL-1 causing cytokine storm in COVID-19: A promising inhibitory strategy. J. Biol. Regul. Homeost. Agents.

[43] Conti P., Caraffa A., Gallenga C., Kritas S.K., Frydas I., Younes A., Di Emidio P., Tetè G., Pregliasco F., Ronconi G. (2021). The British variant of the new coronavirus-19 (Sars-Cov-2) should not create a vaccine problem. J. Biol. Regul. Homeost. Agents.

[44] Oladunni F.S., Park J.-G., Pino P.A., Gonzalez O., Akhter A., Allue-Guardia A., Olmo-Fontanez A., Gautam S., Garcia-Vilanova A., Ye C. (2020). Lethality of SARS-CoV-2 infection in K18 human angiotensin-converting enzyme 2 transgenic mice. Nat. Commun..

[45] Costela-Ruiz V.J. (2020). SARS-CoV-2 infection: The role of cytokines in COVID-19 disease. Cytokine Growth Factor Rev..

[46] DeDiego M.L. (2014). Inhibition of NF-kappaB-mediated inflammation in severe acute respiratory syndrome coronavirus-infected mice increases survival. J. Virol..

[47] Nieto-Torres J.L., DeDiego M.L., Verdiá-Báguena C., Guardeno J.M.J., Regla-Nava J.A., Fernandez-Delgado R., Castaño-Rodriguez C., Alcaraz A., Torres J., Aguilella V.M. (2014). Severe Acute Respiratory Syndrome Coronavirus Envelope Protein Ion Channel Activity Promotes Virus Fitness and Pathogenesis. PLoS Pathog..

[48] Siu K.-L., Yuen K.-S., Castano-Rodriguez C., Ye Z.-W., Yeung M.-L., Fung S.-Y., Yuan S., Chan C.-P., Yuen K.-Y., Enjuanes L. (2019). Severe acute respiratory syndrome Coronavirus ORF3a protein activates the NLRP3 inflammasome by promoting TRAF3-dependent ubiquitination of ASC. FASEB J..

[49] Conti P., Ronconf G., Caraffa A., Gallenga C.E., Ross R., Frydas I., Kritas S.K. (2020). Induction of pro-inflammatory cytokines (IL-1 and IL-6) and lung inflammation by Coronavirus-19 (COVI-19 or SARS-CoV-2): Anti-inflammatory strategies. J. Biol. Regul. Homeost. Agents.

[50] Chen J. (2010). Cellular immune responses to severe acute respiratory syndrome coronavirus (SARS-CoV) infection in senescent BALB/c mice: CD4+ T cells are important in control of SARS-CoV infection. J. Virol..

[51] Mahajan K., Mahajan N.P. (2012). PI3K-independent AKT activation in cancers: A treasure trove for novel therapeutics. J. Cell. Physiol..

[52] Mahajan K., Coppola D., Challa S., Fang B., Chen Y.A., Zhu W., Lopez A.S., Koomen J., Engelman R.W., Rivera C. (2010). Ack1 Mediated AKT/PKB Tyrosine 176 Phosphorylation Regulates Its Activation. PLoS ONE.

[53] Joung S.M., Park Z.-Y., Rani S., Takeuchi O., Akira S., Lee J.Y. (2010). Akt Contributes to Activation of the TRIF-Dependent Signaling Pathways of TLRs by Interacting with TANK-Binding Kinase 1. J. Immunol..

[54] Chen R., Kim O., Yang J., Sato K., Eisenmann K.M., McCarthy J., Chen H., Qiu Y. (2001). Regulation of Akt/PKB Activation by Tyrosine Phosphorylation. J. Biol. Chem..

[55] Jiang T., Qiu Y. (2003). Interaction between Src and a C-terminal Proline-rich Motif of Akt Is Required for Akt Activation. J. Biol. Chem..

[56] Reddy N.M., Potteti H.R., Vegiraju S., Chen H.-J., Tamatam C.M., Reddy S.P. (2015). PI3K-AKT Signaling via Nrf2 Protects against Hyperoxia-Induced Acute Lung Injury, but Promotes Inflammation Post-Injury Independent of Nrf2 in Mice. PLoS ONE.

[57] Li L.-F., Liu Y.-Y., Yang C.-T., Chien Y., Twu N.-F., Wang M.-L., Wang C.-Y., Huang C.-C., Kao K.-C., Hsu H.-S. (2013). Improvement of ventilator-induced lung injury by IPS cell-derived conditioned medium via inhibition of PI3K/Akt pathway and IP-10-dependent paracrine regulation. Biomaterials.

[58] Kalil A.C., Patterson T.F., Mehta A.K., Tomashek K.M., Wolfe C.R., Ghazaryan V., Marconi V.C., Ruiz-Palacios G.M., Hsieh L., Kline S. (2021). Baricitinib plus Remdesivir for Hospitalized Adults with Covid-19. N. Engl. J. Med..

[59] Song M., Bode A.M., Dong Z., Lee M.-H. (2019). AKT as a Therapeutic Target for Cancer. Cancer Res..

[60] Dorlo T.P.C., Balasegaram M., Beijnen J.H., De Vries P.J. (2012). Miltefosine: A review of its pharmacology and therapeutic efficacy in the treatment of leishmaniasis. J. Antimicrob. Chemother..

[61] National Research Council (U.S.), Committee for the Update of the Guide for the Care and Use of Laboratory Animals, Institute for Laboratory Animal Research (U.S.), National Academies Press (U.S.) (2011). Guide for the Care and Use of Laboratory Animals.

