# Supplementary material for: The Pro-Inflammatory Chemokines CXCL9, CXCL10 and CXCL11 Are Upregulated Following SARS-CoV-2 Infection in an AKT-Dependent Manner
Source: Viruses. 2021 Jun 3;13(6):1062. doi: 10.3390/v13061062 (PMC8226769; doi:10.3390/v13061062)
Supplement: Supplementary file 1 [file viruses-13-01062-s001.zip › viruses-1230210-supplementary.pdf]

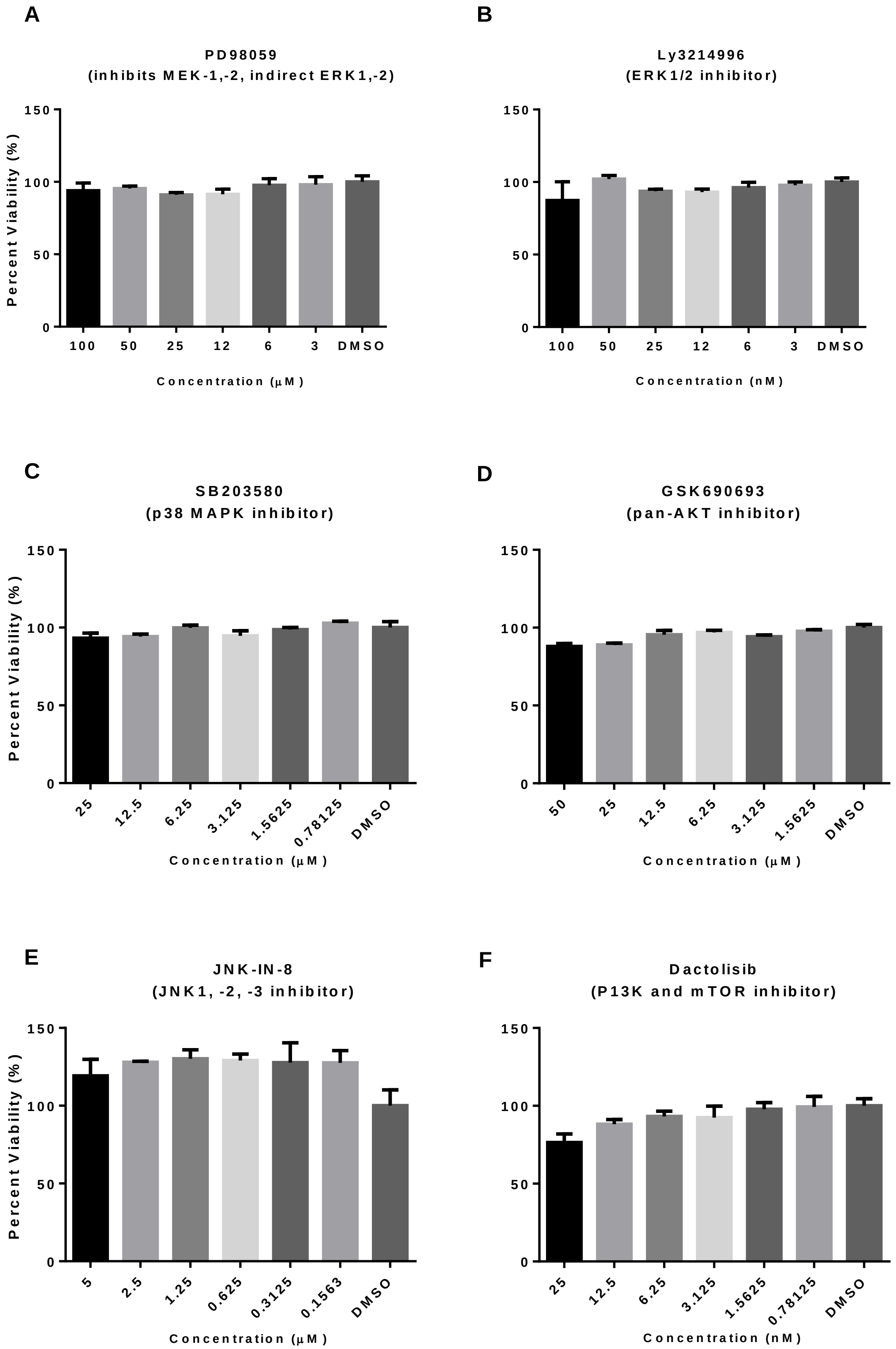

Figure S1. Cytotoxicity of kinase inhibitors in Calu-3 cells. Calu-3 cells were exposed to the indicated kinase inhibitors, over a range of concentrations. Percent cellular viability was determined at 24 h post-treatment using CellTiter Glo. The percent viability (relative luminescence units; RLU) of treated cells, and those exposed to vehicle (DMSO) alone, were normalized to untreated cells. Data represent the mean ± SEM (n=3).
